# HER/ErbB receptor interactions and signaling patterns in human mammary epithelial cells

**DOI:** 10.1186/1471-2121-10-78

**Published:** 2009-10-31

**Authors:** Yi Zhang, Lee Opresko, Harish Shankaran, William B Chrisler, H Steven Wiley, Haluk Resat

**Affiliations:** 1 Computational Biology and Bioinformatics Group, Pacific Northwest National Laboratory, Richland, WA, 99352, USA; 2 Department of Medicine, University of Tennessee Health Science Center, Memphis, TN, 38103, USA; 3 Cell Biology and Biochemistry Group, Pacific Northwest National Laboratory, Richland, WA, 99352, USA; 4 Environmental Molecular Sciences Laboratory, Pacific Northwest National Laboratory, Richland, WA, 99352, USA

## Abstract

**Background:**

Knowledge about signaling pathways is typically compiled based on data gathered using different cell lines. This approach implicitly assumes that the cell line dependence is not important. However, different cell lines do not always respond to a particular stimulus in the same way, and lack of coherent data collected from closely related cellular systems can be detrimental to the efforts to understand the regulation of biological processes. To address this issue, we created a clone library of human mammary epithelial (HME) cells that expresses different levels of HER2 and HER3 receptors in combination with endogenous EGFR/HER1. Using our clone library, we have quantified the receptor activation patterns and systematically tested the validity of the existing hypotheses about the interaction patterns between HER1-3 receptors.

**Results:**

Our study identified HER2 as the dominant dimerization partner for both EGFR and HER3. Contrary to earlier suggestions, we find that lateral interactions with HER2 do not lead to strong transactivation between EGFR and HER3, i.e., EGFR activation and HER3 activation are only weakly linked in HME cells. We also find that observed weak transactivation is uni-directional where stimulation of EGFR leads to HER3 activation whereas HER3 stimulation does not activate the EGFR. Repeating our experiments at lower cell confluency established that cell confluency is not a major factor in the observed interaction patterns. We have also quantified the dependence of the kinetics of Erk and Akt activation on different HER receptors. We found that HER3 signaling makes the strongest contribution to Akt activation and that, stimulation of either EGFR or HER3 leads to significant Erk activation.

**Conclusion:**

Our study shows that clone cell libraries can be a powerful resource in systems biology research by making it possible to differentiate between various hypotheses in a consistent cellular background. Using our constructed clone library we profiled the cell signaling patterns to establish the role of HER2 in the crosstalk between EGFR and HER3 receptors in HME cells. Our results for HME cells show that the weak linkage between EGFR and HER3 pathways can lead to distinct downstream cellular signaling patterns in response to the ligands of these two receptors.

## Background

The human epidermal growth factor receptors (HER, also known as ErbB) belong to the receptor tyrosine kinase superfamily. They are key regulators of physiological processes such as cell proliferation, survival, and migration [1]. The first three members of this receptor family, EGFR/HER1, HER2 and HER3, play important roles in carcinogenesis, and they are often co-expressed [2,3]. HER receptors are highly homologous and their activation occurs through similar biochemical steps, although certain properties of the receptor members are distinct. For example, HER2 receptors have no known ligand and HER3 lacks intrinsic tyrosine kinase activity [4,5], and upon ligand binding, they all undergo conformation changes that favor the formation or stabilization of receptor hetero- and homo-dimers [6,7]. This results in receptor phosphorylation, and the resultant formation of complexes with adaptor proteins to initiate signaling. This cascade of events leads to downstream signal transduction, and can trigger diverse biological responses [8-10].

Almost every possible pairwise combination of HER dimers has been reported [11]. Ligand availability [12], the cellular repertoire of these HER receptors [13], and the receptor dimerization hierarchy [14,15] are the major determinants of the dimer formation kinetics. Each HER receptor appears to have a characteristic repertoire of adaptor proteins depending on its dimerization partner [16]. Thus, the types of receptor homo- and hetero-dimers that are formed, and the signal transduction pathways that are activated depend upon the specifics of the system and treatment conditions. This severely complicates the modeling of the HER signaling pathways and the investigation of the HER-initiated cellular responses [17-20].

Biomedical and biomolecular studies have shown that inhibition of HER dimerization and HER-mediated signaling can be an extremely effective therapeutic strategy [21]. In HER2 positive breast cancers, heterodimer interactions between HER2 and its partners are often constitutively activated and their disruption has proven to be an effective means for inhibiting HER2-mediated aberrant responses [1,22]. HER3 overexpression has also been shown to correlate with poor prognosis in epithelial cancers [23,24]. Activation of pro-survival responses through the HER3 pathway can also lead to drug resistance in cancer treatment [25,26]. Further, interactions between members of the HER family and signaling redundancies can also contribute to drug resistance when specific inhibitors are used against a single HER receptor [21,25,27]. Thus, the use of combination therapeutics that target multiple members of the HER family of receptors is advocated as an effective treatment strategy for a variety of tumors [25,26,28,29].

In terms of downstream elements, HER receptors activate the MAPK/MEK/Erk and PI3K/PKB/Akt signaling pathways [1,8], which are thought to be the general mitogenic and pro-survival pathways, respectively. Many stimuli, including growth factors, cytokines, ligands for G protein-coupled receptors, transforming agents, and carcinogens, activate the Erk (extracellular signal-regulated kinase) pathway. Sustained Erk activation can have significant functional consequences [30,31]. For example, constitutive or prolonged Erk activation through HER receptors contributes to speedy cell migration [32] and enhances cell transformation and resistance to apoptosis [33]. The serine/threonine kinase Akt promotes cellular survival by blocking apoptosis, which it achieves by binding and regulating many downstream effectors; e.g., NF-κB, Bcl-2 family proteins, and MDM2 [34]. Akt also induces protein synthesis and therefore plays a role in tissue growth. Akt has been implicated in many types of cancers because of its possible role in angiogenesis and tumor development [35,36]. In particular HER3 is known to have a unique ability to efficiently engage the pro-survival PI3K/Akt pathway [37,38].

**Figure 1:**
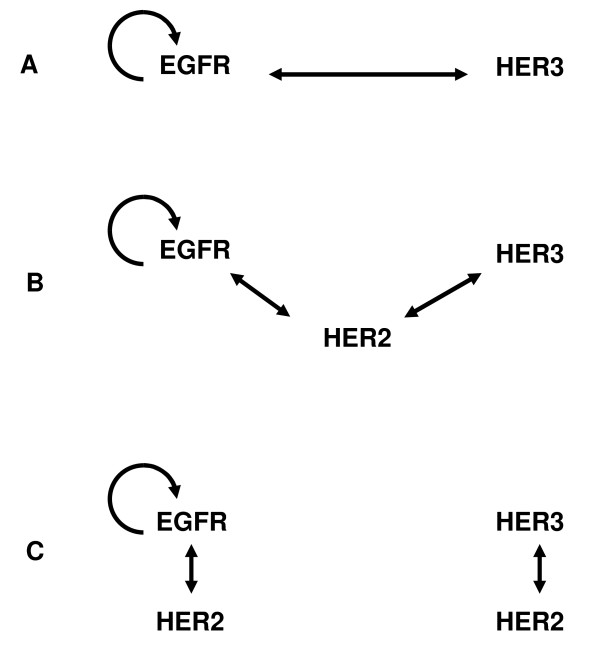
**Schematic diagrams for hypothesized HER receptor interaction patterns**. These diagrams illustrate the EGFR-HER3 interaction patterns that have been reported in the literature. (A) EGFR and HER3 receptors interact directly, i.e., HER2 is not required; (B) HER2 is the explicit facilitator of a lateral interaction; and (C) EGFR and HER3 do not interact with each other but both of them independently interact with HER2. EGFR can also activate itself by forming homodimers.

As summarized above, many aspects of HER signaling have been well examined over the years. However, the body of knowledge has been constructed from in vitro studies that use different cell lines and treatment conditions. Unfortunately, the characteristics of cellular signaling pathways can be cell line- and condition-dependent, as evidenced by conflicting reports and incomplete understanding about particular aspects of the HER system. For example, earlier studies have unquestionably established that HER2 plays a key role in HER signaling. However, how HER2 functions is still uncertain after numerous studies. In particular, it is not clear whether HER2 is merely a preferred dimerization partner of other HER receptors or whether it mediates lateral information transfer between them [13]. Furthermore, the role of HER2 seems to be different in different cells lines and under different conditions [14]. The existing ideas about the interaction mechanisms between the HER receptors [13], and the role of HER2 in facilitating the interaction between EGFR and HER3 can be summarized in the form of the distinct hypotheses illustrated in Figure 1. Briefly, (A) EGFR and HER3 receptors interact directly, i.e., HER2 is not required; (B) HER2 is the explicit facilitator of a lateral interaction; and (C) EGFR and HER3 do not interact with each other but both of them independently interact with HER2. In addition to the heterodimerization-mediated activation of receptors, EGFR can also be activated through the formation of homodimers. However, HER2, which does not bind ligand, and HER3, which is kinase inactive, are only activated through heterodimerization.

These conflicting observations about the possible role of HER2 illustrate the context dependence of cellular signaling. Hence, in order to understand the interactions between the HER molecules under physiological and pathological conditions, we not only need to study systems expressing different levels of these receptors, but also need to do so in a common cellular background to ensure internal consistency of the generated datasets. This is the main objective of our study: We have developed a clone library of human mammary epithelial (HME) cell lines that express endogenous levels of EGFR and varying levels of HER2 and HER3 to experimentally examine the various hypothesized roles of HER2 in coupling EGFR and HER3 activation (Figure 1). Results of our systematic investigation indicate that, although HER2 is the preferred dimerization partner for both the EGFR and HER3, it only weakly couples EGFR and HER3 activation in HME cells. HER2 plays a significant role in the activation of EGFR and HER3 in response to their respective ligands, but the presence of HER2 does not lead to strong crosstalk (transactivation) between EGFR and HER3 receptors. We also find that the observed weak coupling is largely unidirectional, i.e., EGFR ligands can induce HER3 activation in the presence of HER2 but the stimulation of the HER3 pathway by its ligand does not induce EGFR activation regardless of the presence of HER2. The receptor activation patterns reported here provide us with information on the HER interaction hierarchy in HME cells.

The second objective of our study is to investigate how the changes in the activation levels of HER1-3 receptors alter the phosphorylation patterns of downstream effectors Erk and Akt upon ligand stimulation. In parallel experiments using the developed clone cell library, EGFR and HER3 were stimulated using their respective ligands EGF and heregulin (HRG), and Erk and Akt phosphorylation levels were measured. Our results indicate that HER3 activation is the strongest inducer of Akt activation in HME cells. We also observe that, even though it is at a lower level compared to stimulation through the EGFR, Erk activation occurs at significant levels upon HER3 stimulation. Even though the observed Akt phosphorylation pattern is similar to earlier measurements using the MCF-7 human breast cancer cell line [19], Erk activation patterns in HME cells are different than in MCF-7 cells [19]. This yet again shows that a comprehensive investigation of the HER signaling system in a consistent cellular background is warranted.

We note that, unlike many model cell lines with limited physiological responses to growth factors, HME cells are an excellent model system for studying the properties of the HER system because, like many epithelium derived cell types, HME cells require EGFR activation for proper proliferation and migration responses [39]. The 184A1 strain of HME cells that was used to develop our clone library retains the EGFR-dependent regulatory machinery of the primary cell type from which it was derived [40]. Thus, the data obtained in our study provide relevant information for developing directed and effective medical treatments for aberrant responses in epithelial cells.

## Methods

Epidermal growth factor (EGF, human recombinant) and heregulin-β1 (HRG, human recombinant) were purchased from Peprotech (Rocky Hill, NJ). All ELISA assay kits were purchased from R&D Systems Inc. (Minneapolis, MN). Humanized monoclonal antibodies 7C2, 2C4 (Pertuzumab) and 4D5 (Herceptin, Trastuzumab) were generous gifts from Genentech, Inc. (South San Francisco, CA). All the other reagents were from Sigma (St. Louis, MO) unless otherwise indicated.

### Generation of HME Cell Lines

The human mammary epithelial (HME) cell line 184A1L5 was initially provided by Martha Stampfer (Lawrence Berkeley Laboratory, Berkeley, CA) and maintained at 37°C in 5% CO_2_/air in DFCI-1 medium supplemented with 12.5 ng/ml EGF as described in [41]. This parental cell line expresses high levels of EGFR and relatively low copy numbers of HER2 and HER3 and is labeled the HER2-/3- cell line in our study.

This parental cell line was used to create the HER2 overexpressing cell line by retroviral transduction, as described elsewhere [42]. Briefly, the retroviral MFG vector containing HER2 was constructed and transfected into the ψ CRIP packaging cell line [43-45]. The transfected clones with highest level of HER2 expression were expanded to produce viral supernatant. This supernatant was then used to infect the HME cells. Individual clones of retrovirus transduced HME cells were isolated using cloning rings. The degree of HER2 overexpression was determined by immunofluorescence, flow cytometry, and equilibrium-binding studies using labeled Fab fragments. Growth medium for the HER2 expressing cells was the same as that of the parental HME cells except for addition of the antibiotic G418 (250 μg/ml; Invitrogen, Carlsbad, CA) to ensure selection.

HER3 expressing cells were derived in a similar retrovirus-based strategy. Retroviral pBM-IRESpuro vector containing HER3 and the puromycin resistance gene was transfected into the Phoenix packaging cells [46]. Viral supernatant was collected from transiently transfected Phoenix cells. The supernatant was then used to infect the parental HME cells and the HER2 expressing cells (discussed in the previous paragraph) to create the HER3 expressing and HER2-HER3 coexpressing cell lines, respectively (Table 1). The cell lines expressing HER3 were then grown in the DFCI-1 medium with the addition of 2 μg/ml puromycin (Sigma, St. Louis, MO).

**Table 1:** Receptor expression levels in HME cell lines

**Clone**	**Order of gene transfer**	**Drug selection**	**EGFR (10^3^/cell)**	**HER2 (10^3^/cell)**	**HER3 (10^3^/cell)**
HER2-/3-	Parental	none	200^a ^± 8	30^b^	2^c, d^
HER2+/3-	HER2 into HER2-/3-	G418	151^b ^± 5	600^a ^± 19	2^c, d^
HER2-/3+	HER3 into HER2-/3-	Puromycin	200^b ^± 7	30^b^	28 ± 1^c^
HER2+/3+	HER3 into HER2+/3-	G418 and Puromycin	84^b ^± 7	625^b ^± 34	28 ± 3^c^

^a ^An EGFR expression level of 200 K for the HER2-/3- cell line and a HER2 expression level of 600 K for HER2+/3- cell line were reported previously [42].

^b ^These EGFR and HER2 expression levels were calculated by normalizing the receptor masses measured in ELISA experiments with the respective EGFR and HER2 expression levels shown in item (a) above.

^c ^These HER3 expression levels were calculated by normalizing the receptor masses measured in ELISA experiments with the EGFR receptor expression level shown in item (a) above. Detected expression values were validated by re-computing them based on relative readings with respect to HER2 expression.

^d ^This is also roughly the detection limit in our ELISA experiments.

The stability of clone cell lines over growth passages was consistently monitored, and they were stable for at least 20 passages. Still, in our study, only the cells at low passages were utilized to generate experimental data, and cells were maintained under G418 and puromycin selection as appropriate to ensure transduction efficiency. We note that the parental cell line refers to the original 184A1L5 cells, which were not mock transfected by an empty vector. We also note that we use the term "cell library" to refer to our clone library of sub-lines of this parental HME cell type.

The abundances of HER receptors on all the generated clones were characterized using flow cytometry, with Alexa-488 conjugated mAb 7C2 against HER2 and PE conjugated anti-HER3 antibody (R&D Systems Inc., Minneapolis, MN) against HER3, respectively. Expression levels of HER receptors were further confirmed by ELISA assays.

### Cell Activation

When cells grew to near confluency, regular culture medium was replaced with DFCI-1 medium lacking all supplements but 0.1% bovine serum albumin. Cells were then quiescenced for 12-18 hours before stimulation. Cells were activated through HER receptors by adding either 12 ng/ml EGF or 40 ng/ml HRG, or both, into the culture medium. Activated cells were incubated at 37°C for fixed amounts of time between 0 to 1 hours. Samples were then collected by cooling cells down to 4°C on ice and lysing cells using lysis buffer. In the experiments with antibody blocking, 10 μg/ml antibody (2C4 or 4D5) was preincubated with cells for four hours before ligand addition. At least two independent measurements were performed, with two biological replicates in each experiment, for every treatment condition. Additional experiments where the cells were grown to ~50% confluency were pursued to investigate the dependence of signaling properties on cell confluency. Culture plates with 50% confluency were obtained by seeding the plates with an initial cell density that was half of those of fully confluent plates.

### Cell Lysis

To lyse the cells and prepare samples for receptor mass and activated/phosphorylated receptor quantification, we solubilized cells with ice cold lysis buffer for 20 min (1% NP-40, 20 mM pH 8.0 Tris buffer, 137 mM NaCl, 10% glycerol, 2 mM EDTA, supplemented with 1 mM heat activated sodium orthovanadate and 1% protease inhibitor cocktail III (Calbiochem, La Jolla, CA)). Cell lysates were collected with a scraper. Lysates were centrifuged at 13,000 rpm for 10 min at 4°C, and the supernatants were transferred into fresh microtubes. Obtained cell lysates were either analyzed immediately or stored at a -80°C freezer until needed.

The steps to prepare cell lysates for detecting Erk and Akt and their activation levels were similar as above, except lysis buffer was replaced with 1 mM EDTA, 0.5% Triton X-100, 5 mM NaF, 6 M urea, 1 mM activated sodium orthovanadate, 1% protease inhibitor cocktail III, 100 μM PMSF and 2.5 mM sodium pyrophosphate, in PBS, pH 7.2-7.4.

### Quantification of receptor mass, receptor phosphorylation, and Erk/Akt activation

The receptor masses and phosphorylation levels of the EGFR/HER2/HER3 receptors, and Erk/Akt phosphorylation levels were quantified in ELISA experiments. R&D DuoSet IC ELISA kits were used in these measurements. Before each ELISA assay, protein concentration of cell lysate was measured using the Bicinchoninic Acid protein quantitation kit (Sigma, St. Louis, MO). The manufacture's protocol was followed in all ELISA measurements except for the assays to detect receptor (EGFR/HER2/HER3) masses (total receptor levels). For the latter, to be able to monitor the reaction dynamically during our measurements, the R&D ELISA protocol was modified. Rather than using streptavidin-HRP to bind a biotinylated detection antibody, we used streptavidin conjugated with alkaline phosphatase (Jackson ImmunoResearch, West Grove, PA) and incubated the assay plate for one hour at 37°C. Afterwards, freshly made 1 mg/ml p-nitrophenyl phosphate substrate in diethanolamine buffer (10 mM diethanolamine, 0.5 mM MgCl_2_, pH9.5) was added to start the reaction. The absorbance from the ELISA reagents was detected immediately using a Molecular Devices microplate reader's kinetic setup at 405 nm for 15 min (Sunnyvale, CA).

## Results

### Expression of EGFR/HER2/HER3 in HME Cells

HER receptor expression was examined in all cell lines using FACS (data not shown). Our previous study indicated an EGFR expression level of 200,000 and a HER2 expression of 20,000 for the parental (HER2-/3-) cell line, and a HER2 expression level of 600,000 for the HER2 overexpressing clone HER2+/3- [42]. Total receptor masses were quantified using ELISA assays in all of our cell lines, and the results were used to estimate the relative receptor expression levels. Table 1 lists the abundances of EGFR/HER2/HER3 in our previously reported (HER2-/3-, HER2+/3-) and newly obtained (a HER3 expresser: HER2-/3+, and a HER2/3 co-expresser: HER2+/3+) cell lines and confirms that we have successfully constructed a series of related HME clones expressing endogenous EGFR and co-expressing either HER2, HER3, or both. This clone cell library allows for the systematic examination of the HER interaction patterns and the impact of different HER dimer types on downstream signaling and cellular responses in HME cells. We note that, although we label HER2-/3- and HER2-/3+ cell lines as HER2 negative these cells express low levels of HER2. Our labeling is for notational convenience, and in our case HER2- corresponds to the endogenous HER2 level and means HER2 was not transduced.

### Receptor activation patterns in HME cell library

We stimulated our cell lines with EGFR ligand (12 ng/ml = 2 nM EGF), with HER3 ligand (40 ng/ml = 5 nM HRG), and with both ligands simultaneously. This enabled us to examine the receptor activation patterns in four cell types stimulated with three different ligand combinations. We chose to use different EGF and HRG concentrations because the response to 2 nM HRG is not as robust as the response to EGF administration at the same dose. Based on the measured binding affinities of these two ligands to their respective receptors [47], the receptor binding ability of 5 nM HRG is comparable to that of 2 nM EGF, thus offering a more sensible comparison of EGFR and HER3 pathway activation.

#### EGFR activation

**Figure 2:**
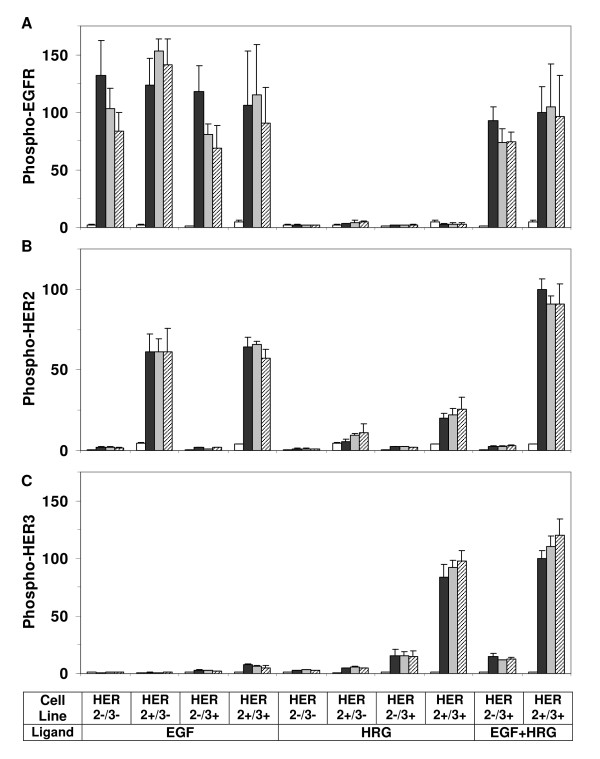
**HER receptor phosphorylation patterns**. Phosphorylation levels of (A) EGFR, (B) HER2, and (C) HER3 are compared between parental cells (HER2-/3-), HER2 expressing cells (HER2+/3-), HER3 expressing cells (HER2-/3+), and HER2-HER3 co-expressing cells (HER2+/3+) during one hour time course after activation with low doses of EGF (12 ng/ml), HRG (40 ng/ml), or both (EGF+HRG). Samples were collected before ligand addition (t = 0), and at t = 10, 30, and 60 min after ligand addition. Receptor phosphorylation levels at these time points are presented as white, black, gray and cross line bars, respectively. Results shown here are the means and standard deviations (SD) of at least four measurements from two biological replicates. The data for each receptor have been normalized with respect to the receptor phosphorylation level of the HER2+/3+ cell line at t = 10 min when both ligands were added.

As shown in Figure 2A, stimulating cells with EGF induced EGFR phosphorylation within minutes of ligand addition in all cell lines. Stimulation of the HER3- cell lines with HRG did not lead to statistically significant EGFR activation. Our data further indicates that:

i) When stimulated with EGF, the presence of HER2 and/or HER3 does not have a major impact on the amplitude of EGFR activation.

ii) The presence of HER2, but not HER3, prolongs EGFR phosphorylation. The difference in phospho-EGFR decay rates between HER2-/3- and HER2+/3- cell lines is noticeable in Figure 2A (p-value = 0.023 when the decay rates are compared). This is consistent with previous reports showing that heterodimer formation with HER2 slows the internalization and degradation of the EGFR thereby prolonging EGFR signaling [48]. The p-value is 0.48 when the decay rates are compared between HER2-/3- and HER2-/3+ cell lines, which indicate that HER3 by itself does not significantly affect EGFR phosphorylation. The effect of HER2 on the EGFR phosphorylation decay is evident even in cells expressing HER3, although this effect is statistically less significant (p-value = 0.092 when HER2-/3+ and HER2+/3+ cells are compared). This indicates that HER2 has a diminished interaction with the EGFR when HER3 is present in the system, most likely due to the competition because HER2 is also the preferred dimerization partner of HER3.

iii) Activation of HER3 by its ligand HRG does not lead to sizable EGFR transactivation. Results for HER2-/3+ and HER2+/3+ cells in Figure 2A show that the stimulation of the HER3 pathway does not directly activate EGFR regardless of the presence of HER2. This observed lack of transactivation supports the notion that while HER2 is the main dimerization partner for the EGFR and HER3 receptors, contrary to earlier suggestions [13], HER2 is not an efficient lateral information carrier between HER receptors in HME cells (Figure 1B).

iv) EGFR phosphorylation when both EGF and HRG are added to the system is comparable to when EGF is added alone (Figure 2A; p-value for the comparison in HER2-/3+ cells is 0.91 and in HER2+/3+ cells is 0.64). This observation is consistent with item (iii) above, and it shows that EGFR does not get transactivated following HER3 activation.

#### HER2 activation

HER2 receptors are activated at high levels after EGF stimulation in all HER2 expressing HME cell lines (Figure 2B), and receptor phosphorylation levels are maintained during the time course of one hour. We have observed a similar dynamic behavior before [17]. This prolonged activation is also observed when HER2 is stimulated through HER3 by addition of HRG. We further note that:

i) Even though the HRG concentration used here is 2.5 times that of EGF and the ligand doses are equivalent when their receptor binding affinities are accounted for (see above), HER2 receptors are activated at a much lower levels through the HRG-stimulated HER3 pathway than through the EGF-stimulated EGFR pathway. However, this might be due to the lower expression level of HER3 in our cell library compared to EGFR (Table 1). When the HER2 phosphorylation results for the HER2+/3+ cell (Figure 2B) in response to EGF and HRG are normalized with respect to the EGFR and HER3 expression levels respectively (EGFR/HER3 ratio = 3, Table 1), we find that the potencies of EGFR and HER3 for inducing HER2 activation are roughly the same. This implies that HER2 phosphorylation in heterodimers with EGFR and HER3 are comparable.

ii) When HER2 is abundant, its activation through the EGFR and HER3 pathways are additive. Comparison of the phospho-HER2 levels in HER2+/3+ cells between EGF+HRG co-stimulation and only EGF addition yields a p-value of 0.001. The p-value for the comparison of HER2 phosphorylation by both ligands EGF+HRG in HER2+/3+ and by EGF activation in HER2+/3- cells is 0.019. As explained below, this suggests that HER2 is the dominant dimerization partner for EGFR and HER3. The receptor activation patterns in the system are determined by the competitive formation of the various dimer types, which is a complex function of the number of available receptors, the dimerization affinities and the stimulus conditions [17,49]. While it is difficult to measure the receptor dimerization patterns, it can be deduced by analyzing the receptor activation profiles in our cell lines. For instance, if EGFR and HER3 preferentially formed dimers with each other (Figure 1A), co-stimulation with EGF and HRG would result in increased phosphorylation of these two receptors rather than a significant increase in HER2 activation. This is clearly not the case in our HME system (Figure 2A-C). In contrast, if HER2 is the dominant dimer partner for EGFR and HER3 (Figure 1C), when there is abundant HER2, we would expect to see additive HER2 activation when EGF and HRG are added simultaneously.

#### HER3 activation

Figure 2C presents HER3 phosphorylation patterns for all the cell lines under different stimulus conditions. Its ligand HRG leads to prominent, prolonged activation of HER3. The most obvious trend in the HRG stimulated phosphorylation of HER3 is its strong dependence on HER2 expression; when HER2 is not present, HER3 phosphorylation is low even when it is directly activated by its own ligand HRG (HER2-/3+ cell line in Figure 2C). This indicates that HER2 is the main and dominant interaction partner for HER3 in HME cells, and this conclusion is further confirmed in our antibody blocking studies (discussed below).

Activation of the system with EGF leads to minimal levels of HER3 phosphorylation. This latter observation further supports the notion that lateral information transfer from EGFR activation to HER3 activation is minimal. Graus-Porta et al have shown that activation of HER3 by EGF can occur via a direct interaction between HER2 and HER3 in T47D and SKBR3 cells [13]. Our results in Figure 2C lend moderate support to this finding: a higher HER2 expression level leads to a slightly higher HER3 activation upon EGF stimulation. However, since HER3 activation is minimal in EGF stimulated HME cells even when HER2 expression level is high, the coupling between the EGFR and HER3 activation is very weak in HME cells. It should be noted that, compared to HER2+/3+ cells, the HER2 expression in HER2-/3+ is very low but this cell line still expresses HER2 (Table 1). Albeit at a low level, this small amount of HER2 can still lead to HER3 phosphorylation when the system is stimulated with HRG (Figure 2C).

### Receptor interactions and transactivation patterns

**Figure 3:**
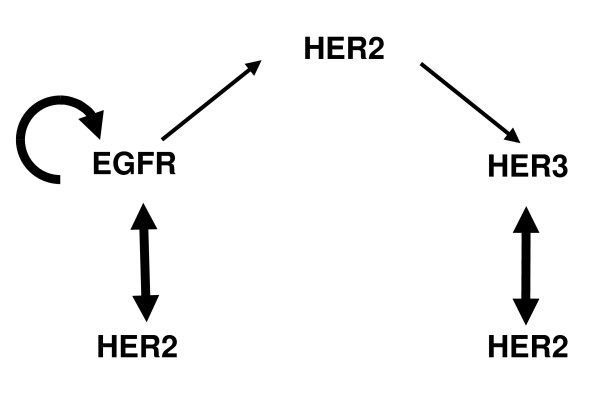
**HER receptor interaction patterns in HME cells**. The receptor interaction patterns in HME cells derived based on our reported results. Thicknesses of the arrows reflect the relative strengths of the interactions.

One of our main aims was to determine which of the existing ideas about the interaction mechanisms between the HER receptors (Figure 1) was valid in HME cells. Our results (Figure 2) show that, with the exception of a low level HER3 activation upon EGF stimulation, the activation of EGFR and HER3 with their respective ligands does not strongly transactivate the other receptor. HER3 is activated at a low level following EGF addition, and this level of activation is slightly enhanced in the presence of HER2 (compare the results for the HER2-/3+ and HER2+/3+ cell lines in Figure 2C). These observations lead us to conclude that diagram A in Figure 1 is not valid for HME cells. The lateral information transfer between EGFR and HER3 through HER2 suggested by Figure 1B does occur at a low level in our system but it is not a major contributor to receptor activation. As both EGFR and HER3 are robustly activated by their own ligands, and a strong phosphorylation of HER2 is observed under all stimulus combinations, diagram 1C is the dominant interaction mechanism between HER receptors in HME cells. Figure 3 summarizes the observed interaction patterns in HME cells. We note that transactivation suggested by diagram 1B occurs in a uni-directional fashion in our system: The presence of HER2 leads to an increase in HER3 phosphorylation when cells are activated with EGF. In contrast, EGFR phosphorylation in response to HRG is almost non-existent irrespective of the HER2 expression level (compare HER2-/3+ and HER2+/3+ cell lines in Figures 2A and 2C). Kim et al. observed that EGF stimulates HER3 phosphorylation in COS7 cells [50]. Tzahar et al. have observed that EGFR coimmunoprecipitates with NDF-activated HER3 in various human tumor cell lines, but the EGFR-HER3 complex was undetectable when cells were stimulated with EGF [14]. In these studies, activation with EGF led to very low levels of HER3 phosphorylation [14]. The differences in EGFR-HER3 transactivation between these previous studies and ours further highlight the cell line dependence of HER interactions, and emphasize the need for a systematic investigation of HER expression and its consequences in a common cellular background.

**Figure 4:**
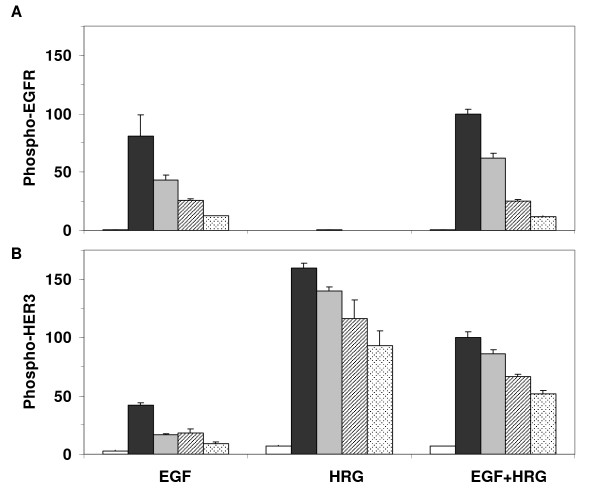
**EGFR and HER3 phosphorylation in response to high ligand doses**. Phosphorylation levels of (A) EGFR and (B) HER3 were measured in the HER3 expressing cell HER2-/3+ during a two hour time course following activation with 100 ng/ml EGF, 80 ng/ml HRG, or both ligands simultaneously. The white, black, gray, cross line and dotted bars represent the receptor phosphorylation levels at t = 0, 10, 30, 60 and 120 min, respectively. Results shown here are the means and SD of two biological replicates. The data in each panel was normalized with respect to the phosphorylation level of the respective receptor at t = 10 min when both ligands were added.

To test whether the lack of observed direct interaction between EGFR and HER3 was due to the strength of the stimuli, we have repeated the experiments using saturating dosages of EGF (100 ng/ml) and HRG (80 ng/ml). These additional experiments with the HER2-/3+ cells produced phosphorylation patterns that were similar to the lower ligand doses (Figure 4), thus supporting our conclusions.

**Figure 5:**
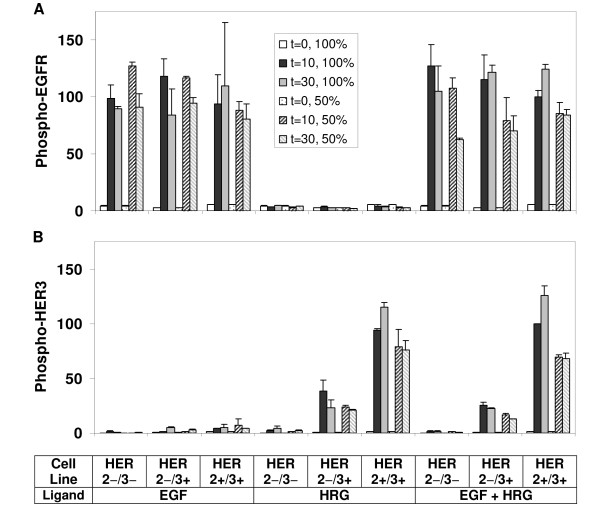
**Effect of cell density on HER receptor phosphorylation patterns**. Phosphorylation levels of (A) EGFR and (B) HER3 are compared between parental cells (HER2-/3-), HER2 expressing cells (HER2+/3-), HER3 expressing cells (HER2-/3+), and HER2-HER3 co-expressing cells (HER2+/3+) after activation with low doses of EGF (12 ng/ml), HRG (40 ng/ml), or both (EGF+HRG) in cells grown to half or full confluency (described in the Methods section). The legend describes the time points and cell confluencies corresponding to each bar in the plot. Results shown here are the means and standard deviations (SD) of two measurements from two biological replicates. The data for each receptor have been normalized with respect to the receptor phosphorylation level of the HER2+/3+ cell line at t = 10 min when both ligands were added.

Data reported in Figure 2 were collected using cultured cells that were grown to full confluency (see Methods section). To test whether cell confluency has an effect on the observed interaction patterns, we repeated some of the experiments using cells grown to 50% confluency. Even though receptor phosphorylation levels are slightly lower in cells grown to half confluency, we find that the transactivation patterns and overall phosphorylation of the EGFR and HER3 receptors are very similar across the clones at full and half confluencies (Figure 5). Thus, cell density does not have a noticeable impact on the HER receptor interaction patterns in HME cells.

We note that our current experiments cannot explain the detailed mechanism of the interactions in diagram 1B, i.e., whether HER2 performs its intermediary role by forming multimeric complexes, or by undergoing two distinct dimerization steps -- first with the EGFR, then with HER3. The former might be applicable to receptor co-localization in lipid rafts [51,52]. The latter, where a receptor dissociates from a dimer and then forms a secondary dimer with a different receptor type, has also been suggested as a possible mechanism for lateral information transfer [13,53]. Whatever the mechanism, we again emphasize that this type of interaction does not seem to play a significant role in HME cells.

### Effects of HER2 dimerization inhibition on receptor activation

Our results shown above and previous studies have clearly illustrated HER2's central role in modulating HER signaling. To further investigate the interaction patterns in this receptor family, and to test the validity of the interaction diagram presented in Figure 3, we used the anti-cancer antibody 2C4 (pertuzumab) to selectively disrupt the ability of HER2 to form dimers. The monoclonal antibody 2C4 is considered to be a general inhibitor of HER2 dimerization because of its ability to bind to the HER dimerization surface [22,54-56]. Since HER2+/3+ cells express all three receptors, we concentrated on this cell line, and used the HER2+/3- and HER2-/3+ cell lines as needed in these validation studies. We predict from our interaction diagram (Figure 3) that 2C4 would significantly lower HER2 phosphorylation under all stimulus conditions. We also expect that EGFR phosphorylation will be partially inhibited in the HER2 expressing cell lines because only the heterodimers with HER2 and not the EGFR homodimers will be blocked. When HER2 expression is too low to play a prominent role (HER2-/3+ cells), EGFR phosphorylation should be left unaffected by 2C4. In contrast, as HER3 mainly forms heterodimers with HER2, its phosphorylation should be significantly decreased in all cases.

**Figure 6:**
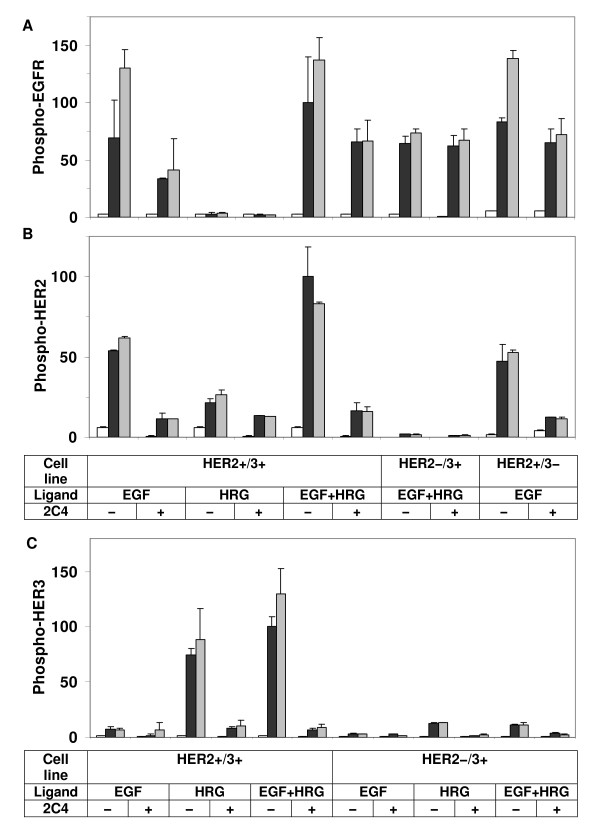
**Inhibition of HER receptor phosphorylation by 2C4**. HER2 blocking antibody 2C4 was used in these experiments to examine the role of HER2 dimerization in the phosphorylation of (A) EGFR, (B) HER2, and (C) HER3. Cells were preincubated with antibody 2C4 for 4 hours before addition of 12 ng/ml EGF, 40 ng/ml HRG, or both. The white, black and gray bars represent the receptor phosphorylation levels at t = 0, 10 and 30 min, respectively. The means and SD of two biological replicates are presented.

Our validation results with the 2C4 antibody are in agreement with these predictions. As predicted, when HER2 is present, 2C4 only partially decreases EGFR phosphorylation (20-50%), while EGFR phosphorylation is not reduced when HER2 is absent (HER2-/3+ cells, Figure 6A). Robust EGFR phosphorylation in the parental HER2-/3- cells (Figure 2A) and the moderate decrease in EGFR phosphorylation in the HER2 expressing cell line upon 2C4 inhibition (Figure 6A) indicate that EGFR homodimers are a major component of EGFR signaling. Although we observe a significant amount of residual phosphorylation in all cases, HER2 phosphorylation was largely (>70%) inhibited by 2C4 in all the cases tested (Figure 6B). We also found that 2C4 decreases HER3 phosphorylation almost down to the detection limit level (>75% inhibition), regardless of the HER2 expression level (Figure 6C). This observation indicates that ligand-induced HER3 activation occurs solely through the formation of HER2-HER3 dimers.

To further investigate the role of HER2-mediated interactions, we used another HER2 blocking antibody, Herceptin [57] in our HER2+/3+ cells. Like 2C4, 4D5/Herceptin also inhibits HER2 dimerization but in a selective fashion; it disrupts the interaction between EGFR and HER2 but does not markedly interfere with the HER2-HER3 interaction [58]. This is most likely due to the differential involvement of HER2 cell surface residues in the formation of the various dimer types [59,60]. Since in our derived interaction diagram (Figure 3) HER3 activation occurs primarily through HER2-HER3 dimerization, we predict that Herceptin should not significantly affect HER3 phosphorylation when HER3 is activated with HRG. We also predict that Herceptin would block HER2 phosphorylation in response to EGF, while minimally affecting HER2 activation in response to HRG. Further, as in the 2C4 case, we expect EGFR phosphorylation to be partially inhibited because only the heterodimers with HER2 will be blocked, and not the EGFR homodimers.

**Figure 7:**
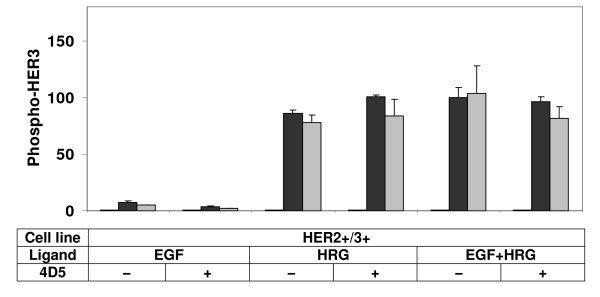
**Inhibition of HER receptor phosphorylation by Herceptin**. HER2 blocking antibody Herceptin/4D5 was used in these experiments to examine the role of HER2 dimerization in the phosphorylation of HER3. Cells were preincubated with antibody Herceptin/4D5 for 4 hours before addition of 12 ng/ml EGF, 40 ng/ml HRG, or both. The white, black and gray bars represent the receptor phosphorylation levels at t = 0, 10 and 30 min, respectively. The means and SD of two biological replicates are presented.

Our results for EGFR and HER2 phosphorylation in the presence of Herceptin matched the above predictions (results not shown). Most importantly, we found that Herceptin did not have a significant effect on HER3 phosphorylation in response to HRG, and to the combined addition of EGF and HRG in the HER2+/3+ cells (Figure 7). These observations lend further support to our conceptual model for HER interactions.

The effect of inhibiting HER2 dimerization on HER3 activation in response to EGF is also presented in Figure 6C and Figure 7, and this data can be used to validate the role of HER2 in EGFR-mediated HER3 transactivation. Based on our interaction diagram (Figure 3), we predict that blocking HER2 should diminish HER3 phosphorylation in response to EGF. Although the HER3 transactivation is low to begin with, inhibiting HER2 dimerization with either 2C4 (Figure 6C) or Herceptin (Figure 7) induces a further decrease in the HER3 activation levels in response to EGF. This confirms the derived role of HER2 in EGFR-mediated HER3 transactivation in HME cells.

### Downstream signaling in response to HER activation

We have also investigated the activation patterns of Erk and Akt in HME cells, which are the primary downstream effectors of the HER family [1,8,37,38]. To characterize the ability of the various HER receptors to activate Erk and Akt we assayed the phosphorylation of these signaling molecules in our clones, which have distinct HER expression profiles.

**Figure 8:**
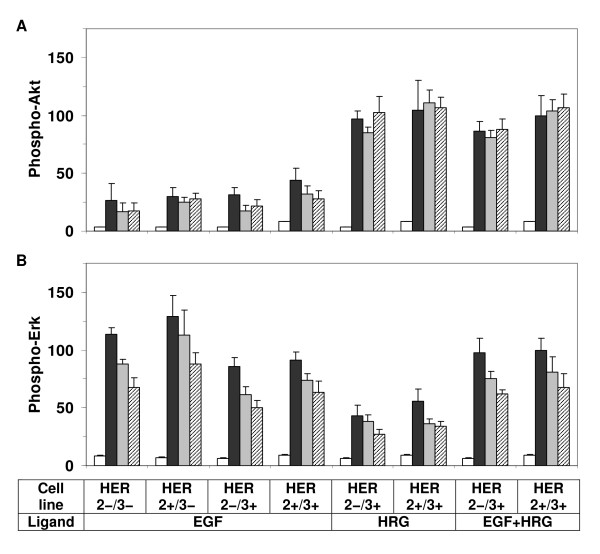
**Akt and Erk activation dynamics**. Akt (A) and Erk1 (B) activation patterns in parental cells (HER2-/3-), HER2 expressing cells (HER2+/3-), HER3 expressing cells (HER2-/3+), and HER2-HER3 co-expressing cells (HER2+/3+) during one hour time course following activation with low dosages of EGF (12 ng/ml), HRG (40 ng/ml), or both (EGF+HRG). Samples were collected at t = 0, 10, 30 and 60 min after ligand addition and receptor phosphorylation levels at these time points are presented as white, black, gray and cross line bars, respectively. Results are presented as the mean and SD of at least four measurements from two biological replicates. The data for each signaling protein was normalized with respect to the phosphorylation level in the HER2+/3+ cell line at t = 10 min when both ligands were added.

HER3 is believed to contribute to pro-survival signaling through Akt activation [26,34]. Therefore, as expected, Akt activation (Figure 8A) correlates well with HER3 phosphorylation in our HME cell lines. We observed a strong Akt response when HER3 was activated by HRG. In contrast, activation of EGFR with EGF leads to a lower Akt response (Figure 8A). A similar pattern has been observed in the MCF-7 cells [19]. However, somewhat surprisingly, Akt was activated at comparable levels in HER2-/3+ and HER2+/3+ cell lines in response to HRG (Figure 2C) even though HER3 phosphorylation levels differed by ~5 fold in these two cells (Figure 8A). Thus, our results indicate that even a low HER3 activation level might be enough to saturate Akt signaling in HME cells. As HER3 signaling mainly occurs through HER2-HER3 dimers (see above), this observation emphasizes the strong potency of this dimer in pro-survival processes. Further, this potency of the HER2-HER3 dimer could explain why HER3 phosphorylation is refractory to tyrosine kinase inhibitors that do not completely silence HER2 [26].

Erk activation was investigated in parallel studies (Figure 8B). Stimulation of the HME cell lines with either EGF or HRG led to immediate robust Erk activation. Comparison of the results for the +HRG and +EGF treatments shows that Erk is more strongly activated by the EGFR pathway than the HER3 pathway. This is opposite of the trend observed in the MCF-7 cells [19]. This however could be due to the unequal EGFR and HER3 expressions and/or due to the mismatch in the activating ligand concentrations between the studies, which further supports our contention that cellular signaling studies have to be pursued in a context dependent manner. Studies have also shown that the Erk pathway can be regulated by PI3K/Akt signaling using different mechanisms. Depending upon the cell type, on the identity of the agonist and the ligand concentration, the Akt upstream signaling protein PI3K may partially contribute to MEK-dependent Erk activation [61]. Akt itself can also inhibit the Erk pathway through its effect on Raf activation [62,63]. Comparison of the +EGF and +EGF+HRG stimulation conditions for the HER3 expressing cells HER2-/3+ and HER2+/3+ shows that Erk activation does not decrease even though Akt activation is enhanced ~3 fold (Figure 8). Thus, substantial crosstalk between Erk and Akt pathways is not apparent in our HME cells.

## Discussion and conclusion

This study reports our results for the receptor activation profiles in a library of HME cells which express HER2 and HER3 receptors at different levels and EGFR/HER1 at an endogenous level. The ability to monitor receptor activation patterns in closely related cell lines has significant advantages. Most importantly, this type of systematic study avoids the ambiguity associated with the interpretation of results collected in different cell lines. As different cell lines may not have the same basic cellular machinery (i.e., their underlying biochemical networks may not be the same), the dynamics of biomolecular processes can show strong cell line dependence. This is evidenced by the contradictory results that often appear in the literature. Further, even large scale cellular phenotypic responses can be different in different cell types. The clone cell library developed in our group provided the means to systematically and quantitatively study the effects of the HER receptor expression profile on signaling patterns in mammary epithelial cells. The library that we have constructed starting with the 184A1 strain of HME cells is physiologically relevant for understanding epithelial cell function because it retains the EGFR-dependent regulatory machinery needed for proper proliferation and migration. Our results reveal that our HME cell library recapitulates several known features of the HER system, lending further evidence to its physiological relevance.

We used measurements of the receptor activation patterns in our cell line library to elucidate the HER interaction hierarchy, which is difficult to directly quantify. Although it is possible to use techniques such as fluorescence resonance energy transfer (FRET) or co-immunoprecipitation (Co-IP) to obtain the relative levels of various dimers, each of these techniques has drawbacks that complicate their interpretation particularly when quantification is desired. FRET requires the use of fluorescent probes or antibodies that could alter the native conformational properties of the receptors. With Co-IP, the pull down efficiencies could be different for the different HER dimer complexes, and transient dimer interactions that result in receptor activation could be missed. Since receptor interactions are required for the activation of the HER molecules, receptor activation patterns represent a convenient readout to establish the interaction hierarchy. However, as evidenced in our discussion of Figures 1 and 3, establishing this link requires careful reasoning because the receptor activation pattern is a complex function of the number of available receptors, the dimerization affinities and stimulus conditions.

In this study we have tested various existing hypotheses about the interactions between HER receptor types in HME cells. We found that, although HER2 is a preferred dimerization partner for both EGFR and HER3, it couples the EGFR and HER3 paths only weakly. These results invalidate the hypothesis that HER2 acts as a lateral information carrier between EGFR and HER3 receptor, a feature that has been observed in some other cell lines. Our analysis reveals that the dominant role of HER2 in HME cells is to amplify EGFR and HER3 signals separately. Based on our observations, we have constructed a conceptual model for HER interactions in HME cells and then validated our model using the HER2 dimerization blocking antibodies 2C4 and Herceptin.

Our observations and the constructed conceptual model for HER receptor mediated signaling in HME cells can be useful in several ways. Conceptual models provide the starting network topology for detailed mechanistic models. Almost all of the existing mechanistic models for the HER family of receptors include EGFR-HER3 dimerization and transactivation (for some recent examples, see [19,20,49]). Depending on the parameter values, the EGFR-HER3 heterodimer may even play a major role in cellular signaling in these models. However, our experimental results have shown that EGFR and HER3 may only weakly affect each other. Thus, a more correct model would exclude the EGFR-HER3 dimerization possibility, at least for our HME cells. Our study clearly allows us to reach relevant conclusions about the network structures necessary for mathematical model building efforts. Another key bottleneck in model building is the lack of consistent, good quality quantitative data for parameter estimation. We have recently shown that extracting kinetic parameters out of the experimental data is not straightforward, and a model-based analysis of such datasets can be a powerful tool for establishing the mechanistic details of the underlying biochemical network for reasonable sized systems [17]. Chen et al. have recently pursued a similar but much more ambitious study [20]. Unfortunately these model building efforts still mix-and-merge kinetic information from multiple studies performed for different cell types under different treatment conditions. Our clone cell library provides us with the ability to generate consistent experimental datasets for mathematical modeling, thereby enabling the quantitative system wide investigation of the critical HER receptor system.

In addition to the interactions between HER members, we have also investigated two signaling pathways that are directly activated by HER receptors, the Akt and Erk signaling pathways, to study the relationship between HER receptor activation and downstream cellular signaling. We found that, even though the HER3 pathway's contribution was comparatively weaker, Erk activation can occur in response to both EGF and HRG stimulation. Our results established HER3 as the main contributor to pro-survival Akt signaling in HME cells. We observed that HER3 activation with HRG is a strong activator of Akt and that even low levels of HER3 mediated signaling might be saturating Akt in HME cells. This could be an indicator for the limited capacity of the Akt pathway, and have other implications. Even though EGF induces low levels of Akt activation, we found that the combined addition of EGF and HRG results in a slight decrease in Akt activation compared to the addition of HRG alone (Figure 7A). Since EGFR and HER3 can activate PI3K/Akt through different recruitment paths [64], when they compete for the limited PI3K/Akt signaling capacity, the amount of Akt phosphorylation reflects the balance between the relative contributions of the EGFR (weak Akt activator) and HER3 (strong activator) pathways. Which receptor ends up controlling Akt activation in a particular cell would depend upon its receptor expression profile and stimulation condition. Given that there is only a weak crosstalk between EGFR and HER3 at the receptor activation level, selectively inhibiting EGFR or HER3 depending upon which route dominates Akt activation could prove to be an effective therapeutic strategy. As we have shown with examples here, results reported in this study can be used to develop and validate new hypothesis about the dynamics of the HER family of receptors and the downstream pathways that they activate.

## Authors' contributions

YZ, HS and HR jointly designed the study and wrote the paper. LO has led the clone library construction effort and helped with writing the paper. HSW has helped with the experimental design. YZ has performed the reported experiments and was helped by WBC. All authors have read and approved the final manuscript.
